# Transcriptome analysis of virulence-differentiated *Fusarium oxysporum* f. sp. *cucumerinum* isolates during cucumber colonisation reveals pathogenicity profiles

**DOI:** 10.1186/s12864-019-5949-x

**Published:** 2019-07-10

**Authors:** Xiao-Qing Huang, Xiao-Hong Lu, Man-Hong Sun, Rong-Jun Guo, Anne D. van Diepeningen, Shi-Dong Li

**Affiliations:** 1 Institute of Plant Protection, Chinese Academy of Agricultural Sciences, Beijing, 100193 China; 2 Wageningen Plant Research, Wageningen University and Research, 6700 AA Wageningen, Netherlands

**Keywords:** *Fusarium oxysporum* f. sp. *cucumerinum*, Cucumber Fusarium wilt, Virulence variation, Differentially expressed genes, Transposon

## Abstract

**Background:**

Cucumber Fusarium wilt, caused by *Fusarium oxysporum* f. sp. *cucumerinum* (*Foc*), is one of the most notorious diseases in cucumber production. Our previous research showed the virulence of *Foc* significantly increases over consecutive rounds of infection in a resistant cultivar. To understand the virulence variation of *Foc* under host pressure, the mildly virulent strain foc-3b (WT) and its virulence-enhanced variant Ra-4 (InVir) were selected and their transcriptome profiles in infected cucumber roots were analyzed at 24 h after inoculation (hai) and 120 hai.

**Results:**

A series of differentially expressed genes (DEGs) potentially involved in fungal pathogenicity and pathogenicity variation were identified and prove mainly involved in metabolic, transport, oxidation-reduction, cell wall degradation, macromolecules modification, and stress and defense. Among these DEGs, 190 up- and 360 down-regulated genes were expressed in both strains, indicating their importance in *Foc* infection. Besides, 286 and 366 DEGs showed up-regulated expression, while 492 and 214 showed down-regulated expression in InVir at 24 and 120 hai, respectively. These DEGs may be involved in increased virulence. Notably, transposases were more active in InVir than WT, indicating transposons may contribute to adaptive evolution.

**Conclusions:**

By a comparative transcriptome analysis of the mildly and highly virulent strains of *Foc* during infection of cucumber, a series of DEGs were identified that may be associated with virulence. Hence, this study provides new insight into the transcriptomic profile underlying pathogenicity and virulence differentiation of *Foc*.

**Electronic supplementary material:**

The online version of this article (10.1186/s12864-019-5949-x) contains supplementary material, which is available to authorized users.

## Background

The *Fusarium oxysporum* species complex contains many destructive fungal plant pathogens. Based on host specificity, the species complex includes more than 150 formae speciales [1], among which *F. oxysporum* f. sp. *cucumerinum* Owen (*Foc*) is notorious for infecting the vascular bundle of cucumber, leading to necrotic lesions on the stem base, foliar wilting, and eventually plant death [2]. *Foc* has been a serious threat to cucumber production around the world [3, 4]. In China, the incidence of cucumber Fusarium wilt is particularly high with a range of 30–90% [5, 6].

Monoculture of resistant germplasm may promote increased selection for virulence and the directed evolution process of the pathogens [7]. In a previous study, a mild virulence isolate, foc-3b, was successively inoculated on resistant and susceptible cucumber cultivars for five generations. The virulence of the original isolate diverged rapidly; virulence was significantly increased after serial passage on the resistant cultivar, especially in the fourth generation, but decreased on the susceptible plants (*p* < 0.05) [8]. This suggests a specific interaction with the plant that selects for enhanced virulence in the pathogen. Understanding the mechanisms of pathogenicity variation of *Foc* is of great importance and necessity to the management of this disease.

Generally, the virulence of a pathogen is mediated by multiple genes in one or more interactive networks [9, 10]. So far, only a few pathogenicity-related genes of *Foc* have been identified, mainly including *fga1*, *fga2* and *fgb1* that encodes G-protein *α* and *β* subunits [11–13] and *FocVel1* that encodes a velvet protein [14]. In addition, several virulence factors of *F. oxysporum* involved in synthesis or regulation of plant cell wall degradation enzymes (CWDEs) and in breaking plant defense have been found to relate to pathogenicity differentiation of *Foc* [8]. Pathogenicity related genes are often encoded in accessory genome regions of *F. oxysporum* and up-regulated during infection [15–18]. Next to pathogenicity genes and effecters, the accessory genomes are enriched in active transposons, and show an accelerated rate of evolution [19, 20], and probably contribute to adaption in response to host pressure.

Transcriptome technology has become a great tool to explore functional genes involved in interactions between plants and pathogens and in pathogenicity differentiation [9, 21–23]. For example, the transcriptomes of Race1 and Race 4, of *F. oxysporum* f. sp. *cubense* that clearly differed in virulence were sequenced in vitro and *in planta*, respectively [9, 21]. During the early infection of banana, pathogenicity genes encoding MAPK, G-proteins, and a two-component system involved in signaling were activated in Race 4 rather than Race 1 [9].When grown in media containing host cell wall polysaccharides, genes involved in penetrating the cell walls of the host, signaling and transportation of nutrients, metabolites, toxic compounds, and some others, were significantly up-regulated in the high virulent strain [21].

In this study, the wild type strain foc-3b (WT) and an induced virulence-enhanced variant Ra-4 (InVir) obtained after four cycles on a resistant cultivar were compared. The comparative transcriptome analysis of those two isolates was conducted to identify virulence-related genes and reveal the progressive virulence evolution of *Foc* under the successive induction of the resistant host. The study provides insight into the mechanisms underlying pathogenicity and virulence differentiation of *Foc* and contributes to the development of new strategies to control plant pathogens.

## Results

### Infection process of *Foc* on cucumber roots

**Fig. 1:**
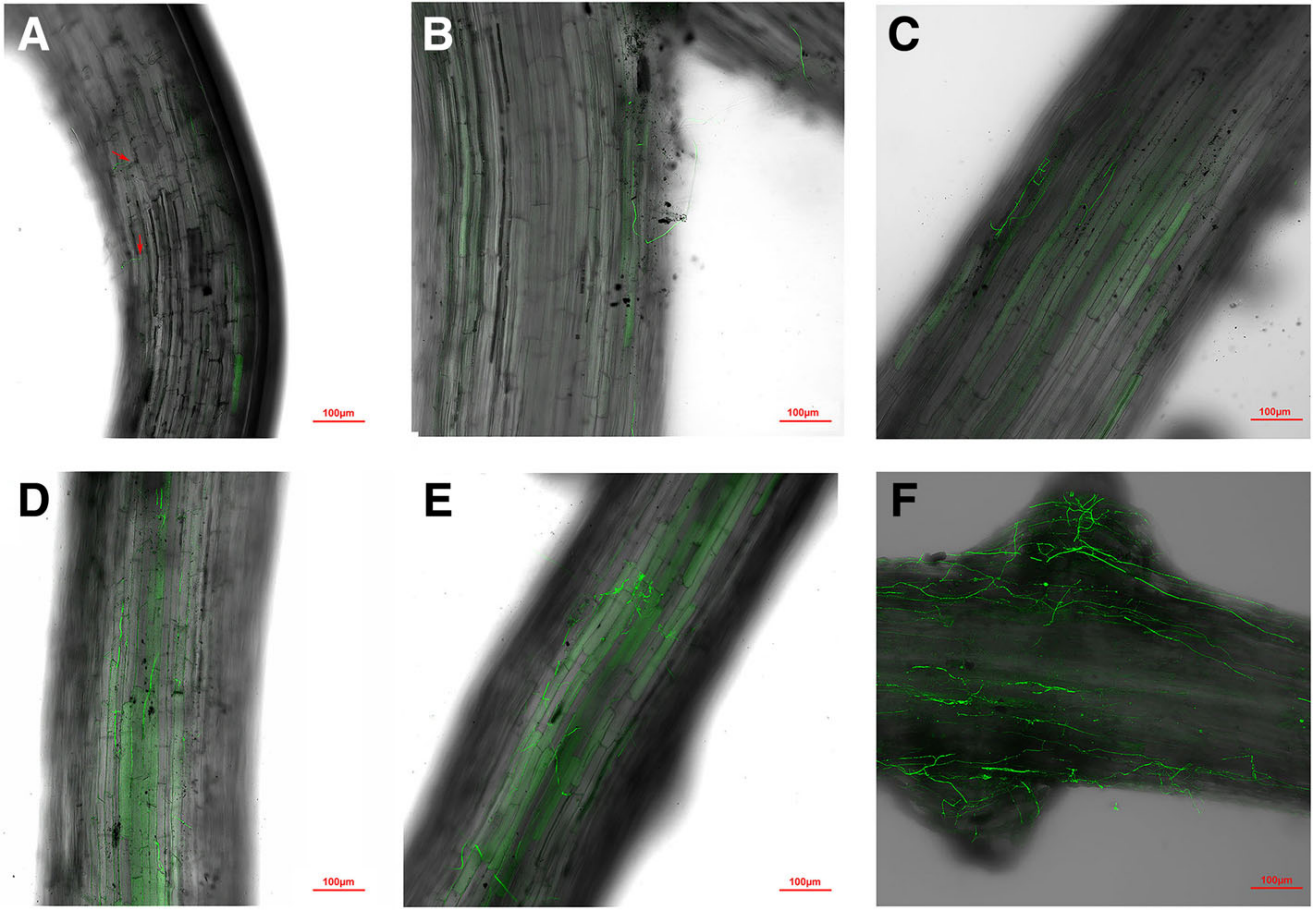
Colonization of cucumber roots by weakly virulent strain WT marked with GFP. Laser scanning confocal microscopy analyses of cucumber roots 24 to 120 h after replanting 7-day-old germinated sterile seedlings in soil containing conidia of transformant gWT. **a** 24 h after inoculation (hai), several spores germinated and begin to penetrate into the plant cell. **b** 36 hai, a small hyphal fragment was observed in the root. **c** 48 hai, **d** 72 hai, hyphae extend along the epidermal and cortical tissue of roots. **e** a small hyphal network has formed in roots 96 hai. **f** 120 hai, hyphae developed in roots and form expanding networks in cucumber roots that exhibits rot symptoms. Arrows represent conidia in root tissue. Scale bars = 100 μm

A transformant gWT was obtained, which constitutively expresses green fluorescent protein (GFP) without affecting fungal growth and its virulence (data not shown). The disease progression in susceptible cucumber cultivar ZN6 was monitored after infection with gWT. Twenty-four hours after inoculation (hai), all plants appeared healthy and few fungal spores were attached to the roots of ZN6, while only some of these spores germinated and began to penetrate into the plant cell (Fig. 1a). At 36 hai, hyphae were loosely attached to the main root surfaces and small hyphal fragments were observed in the root cells (Fig. 1b). Hyphae started to extend along the epidermal and cortical tissue of roots 48 and 72 hai (Fig. 1c-d). A small hyphal network gradually formed 96 hai and several cucumber roots exhibited rot symptoms macroscopically (Fig. 1e). At 120 hai, approximately 60% of the examined plants showed disease symptoms, and hyphae developed and formed expanding networks in cucumber roots (Fig. 1f). The 24 and 120 hai were selected to represent two distinct stages of *in planta* growth after transplanting 7-day-old germinated sterile seedlings in soil containing spores of transformant gWT.

### De novo assembly of the transcriptomes and functional annotation

The transcriptomes of 18 samples of the two isolates consisted of 6 mycelia samples and 12 samples of infected cucumber root tissues were sequenced using the Illumina HiSeq 4000 platform. For each *Foc* strain in vitro, 6 Gb data were generated. Because of the content of *Foc* was very low in the cucumber root tissues, 10–29 Gb data were generated for each *in planta* sample, especially for the mild virulence strain samples. After filtering low quality reads and adaptor sequences, 2.09 billion bp of high quality clean reads remained. The clean data of the six samples of mycelia and spores in vitro were assembled de novo as reference gene set using the Trinity software and 29,806 unigenes, comprising 50,130,317 bp, were obtained, with a mean length of 1,681 bp, a N50 of 3,146 bp and a GC contents of 50.03–50.32% (Additional file 1). Transcriptome completeness, according to BUSCO, ranged between 94.1 and 97.9% (Additional file 2).

Functional annotation of all the unigenes was conducted, and a total of 28,544 unigenes (95.77%) could be annotated in at least one database: 24,604 (82.55%), 24,514 (82.25%), 14,200(47.64%), 11,241(37.71%), 15,524(52.08%) and 11,803 (39.60%) were annotated to the non-redundant (NR), NT, Swiss-Prot, Clusters of orthologous groups (COG), Kyoto encyclopedia of genes and genomes (KEGG), and Gene ontology (GO) databases, respectively. The GO annotation indicated 11,803 unigenes were categorized into 53 functional terms in 3 categories. Among them, genes associated with metabolic, cellular process and single-organism process in the category ‘biological process’; cell and cell part in the category ‘cellular components’; and binding and catalytic activity in the category ‘molecular function’, were the most abundant (Additional file 3). The KEGG pathway database was used to analyze intracellular metabolic processes, and 15,524 unigenes were assigned to 127 KEGG pathways. ‘Metabolic pathways’, ‘biosynthesis of secondary metabolites’, ‘biosynthesis of antibiotics’ and ‘MAPK signaling pathway - yeast’ were the dominant pathways, and the proportions were 28.89, 11.98, 7.8 and 7.18%, respectively (Additional file 4).

### Analysis of DEGs of *Foc*

All clean reads were mapped to the assembled *Foc* reference transcriptome. For the in vitro samples 82.3–87.1% of reads could be mapped to the reference gene set, while for infected root samples, at 24 hai the percentage of reads mapped ranged from 1.12–2.62% for the mild virulence strain WT and 2.28–5.92% for the high virulence strain InVir, and the percentages were raised to 2.09–5.71% and 14.64–32.45% at 120 hai, respectively (Table 1).

**Table 1:** Mapping results of RNA-Seq data from different virulence *Foc* strains in vitro and *in planta* samples

Sample^a^	Clean Data Size (bp)	Clean Reads Number	Total fungal Mapped Reads (%)	Unique fungal Match (%)	Multi-position fungal Match (%)
InVir 0 h-1	6147207600	40981384	87.1	56.31	30.78
InVir 0 h-2	6180869700	41205798	86.25	57.98	28.27
I_0h-3	6169962900	41133086	86.7	58.01	28.69
InVir 24 h-1	10209826200	68065508	4.5	3.3	1.19
InVir 24 h-2	15215636700	101437578	2.28	1.67	0.6
InVir 24 h-3	10211927100	68079514	5.92	4.34	1.59
InVir 120 h-1	10324005300	68826702	31.46	20.8	10.66
InVir 120 h-2	10278412200	68522748	32.45	22.42	10.02
InVir 120 h-3	10328451900	68856346	14.64	10.16	4.49
WT 0 h-1	6060463800	40403092	82.7	55.36	27.34
WT 0 h-2	6062771100	40418474	82.27	53.8	28.47
WT 0 h-3	6104587500	40697250	86.83	57.85	28.99
WT 24 h-1	27172202700	181148018	1.12	0.87	0.25
WT 24 h-2	16219629900	108130866	2.62	1.94	0.68
WT 24 h-3	19779039300	131860262	1.72	1.29	0.43
WT 120 h-1	17173338600	114488924	2.09	1.5	0.6
WT 120 h-2	10163430300	67756202	5.71	3.95	1.76
WT 120 h-3	15385975500	102573170	2.39	1.69	0.71

^a^letters ‘WT’ and ‘InVir’ represent the wild strain foc-3b (lower virulence) and induced virulence variation Ra-4 (high virulence), respectively. The number after the letters represent the sampling times

**Fig. 2:**
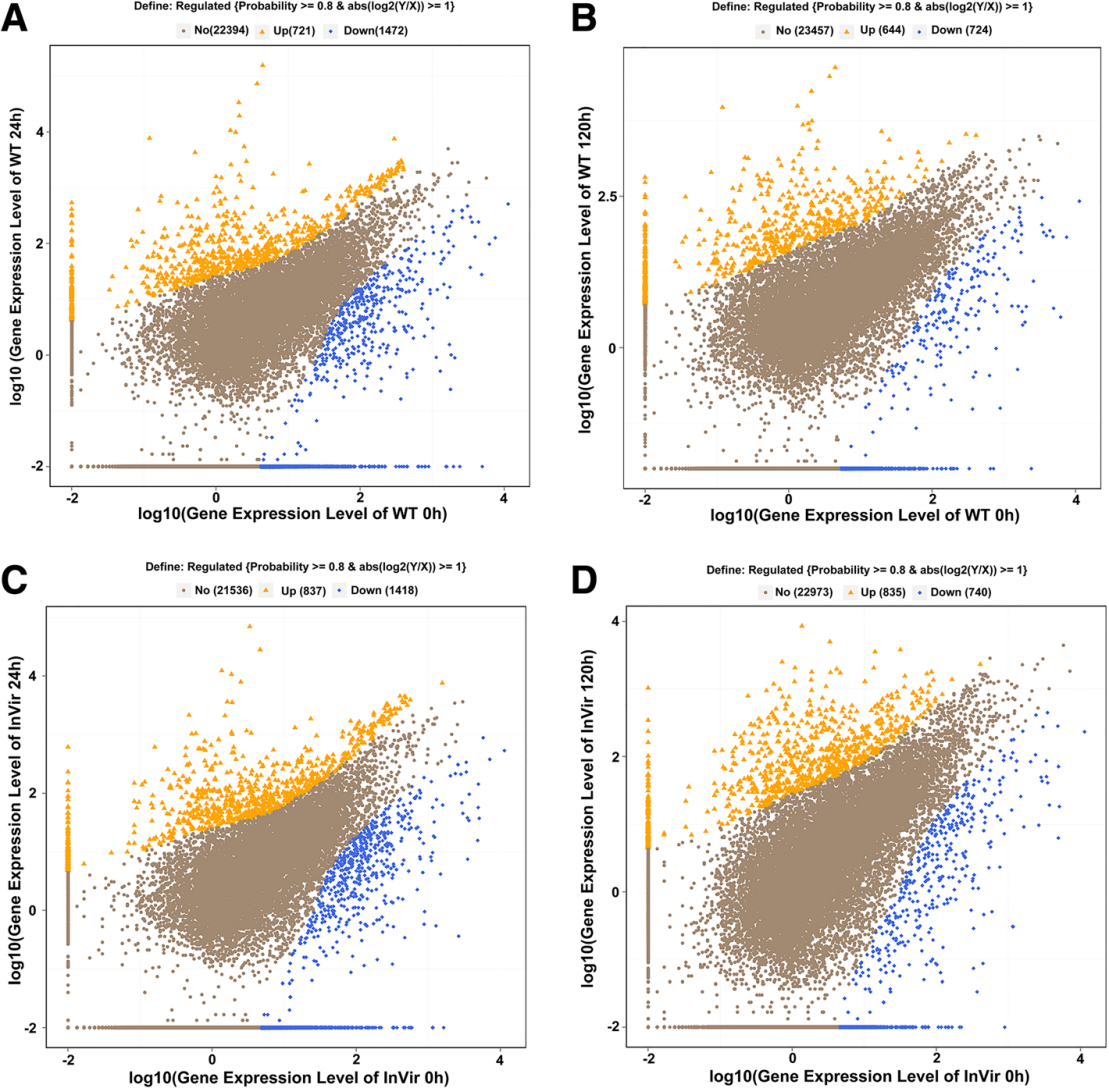
The scatter plots of *Foc* genes differentially expressed between in vitro and *in planta* samples. **a** Differentially expressed genes (DEGs) of lower virulent strain WT compared to vegetative growth at 24 hai. **b** DEGs of WT compared to vegetative growth at 120 hai. **c** DEGs of highly virulent strain InVir at 24 hai. **d** DEGs of InVir at 120 hai. The x and y axes represent log10 gene expression level of in vitro and *in planta*, respectively. The significantly up- and down-regulated genes are presented with orange triangles and blue squares, while non-significant genes as brown dots

During the infection, a total of 7,391 DEGs were detected in the two strains compared to vegetative growth (in vitro) (Fig. 2, Additional file 5). One thousand seventy-three genes were up-regulated in WT, of which 40.0% were only expressed in early stage, and 32.8% were unique to the later infection stage. Of the 721 genes up-regulated at 24 hai, 322 genes showed at least a 64-fold increase in expression levels; 196 showed over 256-fold and 88 showed over 1024-fold increases when compared to germinating conidia and mycelia. Of the up-regulated genes at 120 hai, 431 showed over 64-fold increases; 265 showed over 256-fold and 131 showed over 1024-fold increases (Table 2). Besides, 1,585 genes were found down-regulated, including 611 genes down-regulated at both time points and 861 and 113 specific DEGs at 24 or 120 hai, respectively (Fig. 2). For the highly virulent strain InVir, a total of 1,324 DEGs were up-regulated, among which 489 and 487 DEGs were specific for the early or late infection stage, respectively. Of the 1,324 up-regulated genes, 306 and 396 genes showed over 64-fold induction in infected roots as compared to their corresponding average transcript levels in germinating conidia and mycelia at 24 and 120 hai, respectively (Table 2). Meanwhile 1,534 genes were found down-regulated, of which 624 genes down-regulated at both time points and 794 and 116 specific DEGs for 24 and 120 hai, respectively (Fig. 2).

**Table 2:** Up- or down-regulated *Foc* genes during infection compared to germinating conidia and mycelia

Fold Change^a^	WT 0 vs 24 h		WT 0 vs 120 h		InVir 0 vs 24 h		InVir 0 vs 120 h	
	Up	Down	Up	Down	Up	Down	Up	Down
4	721	1472	644	724	837	1418	835	740
16	521	1309	600	685	508	1187	691	650
64	322	1147	431	587	306	994	392	518
256	196	1069	265	534	183	913	224	401
≥1024	88	387	131	224	74	392	76	153

^a^Fold changes in gene expression during early and late infection stages were calculated by comparing FPKM values of individual genes in infected roots with the average FPKM values of the corresponding genes in spores and mycelia

**Fig. 3:**
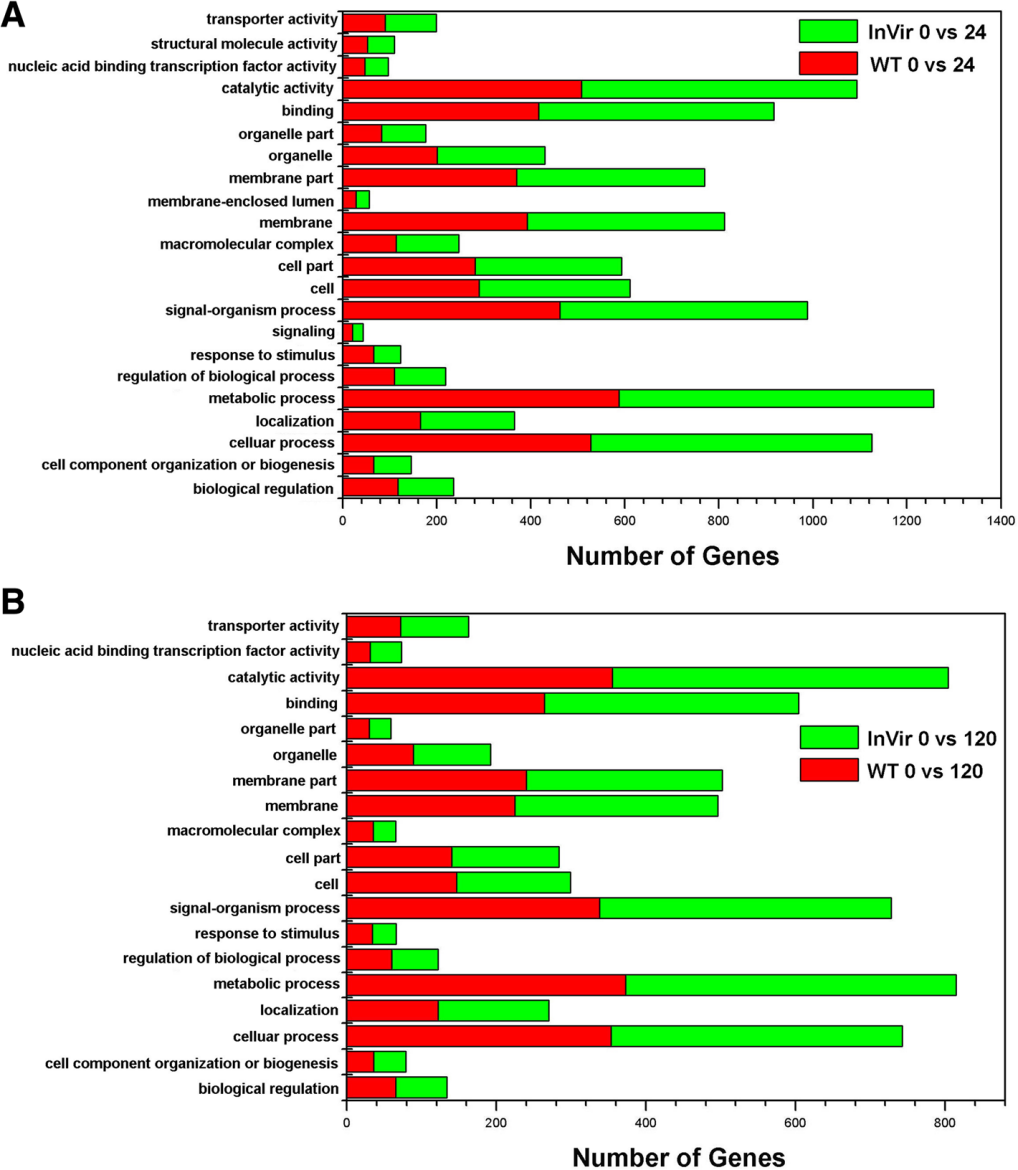
Gene ontology (GO) functional annotation of DEGs *in planta* datasets of *Foc* strains WT and InVir. **a** DEGs of the two strains at 24 hai. **b** DEGs of the two strains at 120 hai. Red and green bars indicate WT and InVir respectively

**Fig. 4:**
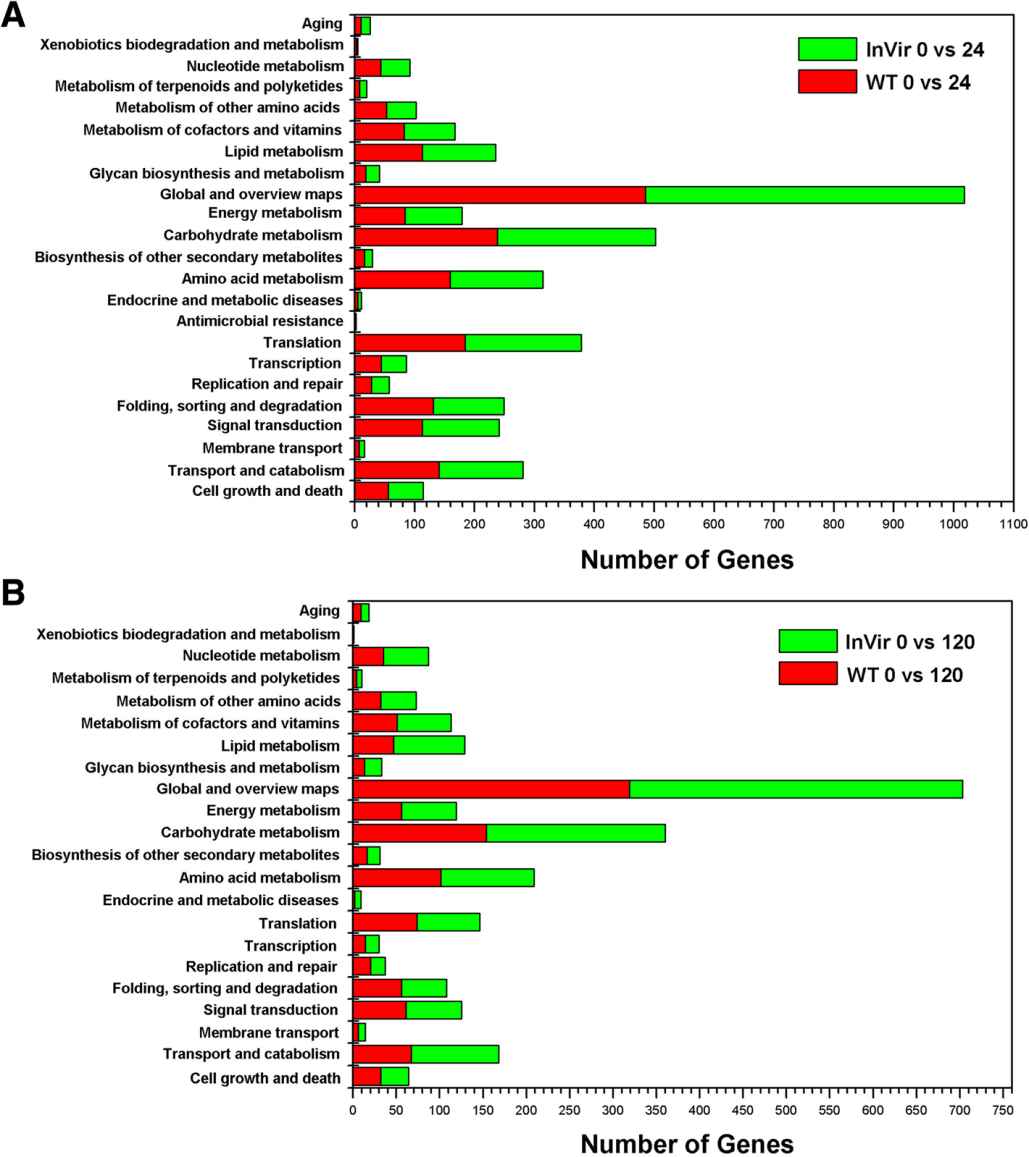
KEGG analysis of the DEGs *in planta* datasets. **a** DEGs of the two strains at 24 hai. **b** DEGs of the two strains at 120 hai. Red and green bars indicate WT and InVir respectively

GO functional classification and KEGG pathway analyses were used to investigate the molecular functions of the identified DEGs. In total 49 and 48 GO terms could be assigned to WT and InVir at 24 hai, respectively. In both strains ‘metabolic process’, ‘membrane’, and ‘catalytic activity’ were the most significant classes in the Biological Process, Cellular Component and Molecular function categories, respectively (Fig. 3a). In the 120 hai datasets, 45 and 43 GO terms were detected in WT and InVir, respectively. The dominant terms were unaltered in InVir, while in WT ‘membrane part’ was the most significant in the Cellular Component category (Fig. 3b). The numbers of DEGs in many GO terms in both isolates were no significant difference at the same time-point after infection (*P* > 0.05). At 24 hai, 120 and 119 metabolism pathways assigned to 23 terms were found in WT and InVir, respectively (Fig. 4a). While at 120 hai, the number of expressed pathways was slightly reduced to 112 and 111, respectively (Fig. 4b). Of these, the metabolic pathway category was most commonly expressed in all datasets, which mainly includes global and overview maps and carbohydrate metabolism terms, followed by pathways involved in genetic information processing and cellular processes. There was no clear difference between the gene numbers of most terms in both isolates.

### Overlapping DEGs in in planta datasets of the two strains link to pathogenicity

**Fig. 5:**
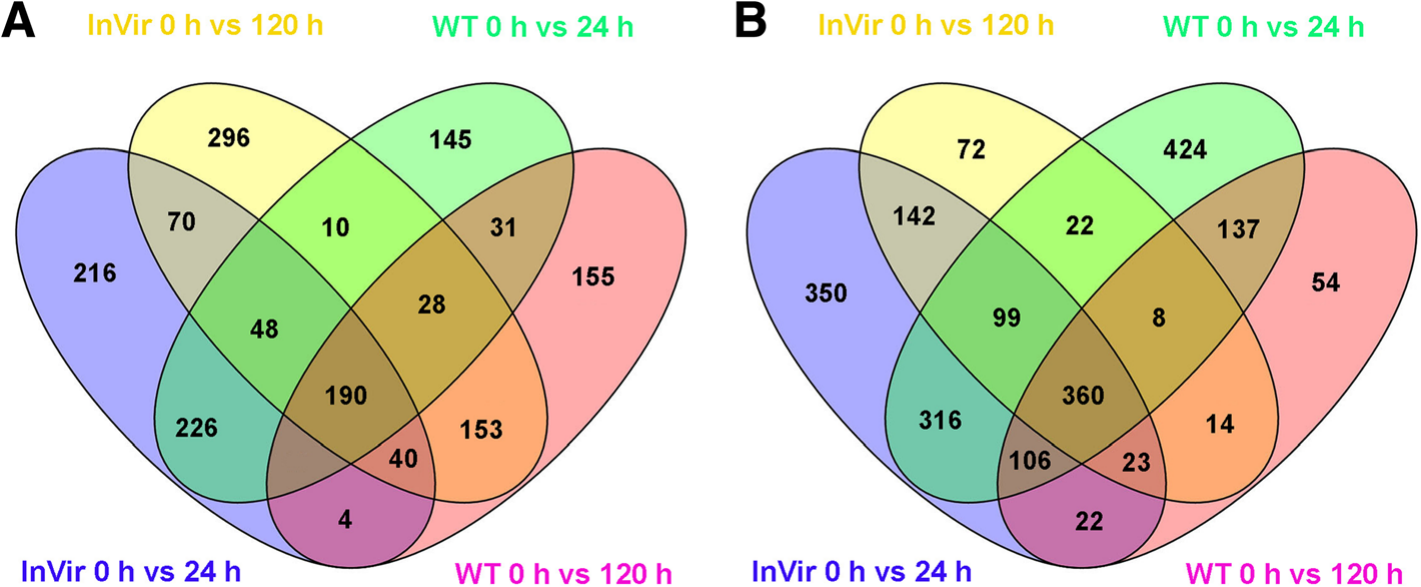
Unique and overlapping Foc DEGs between WT and InVir *in planta* at different time point datasets. **a** Venn diagram of DEGs in overlapping WT and InVir *in planta* up-regulated datasets. **b** Venn diagram of DEGs in overlapping WT and InVir *in planta* down-regulated datasets

**Fig. 6:**
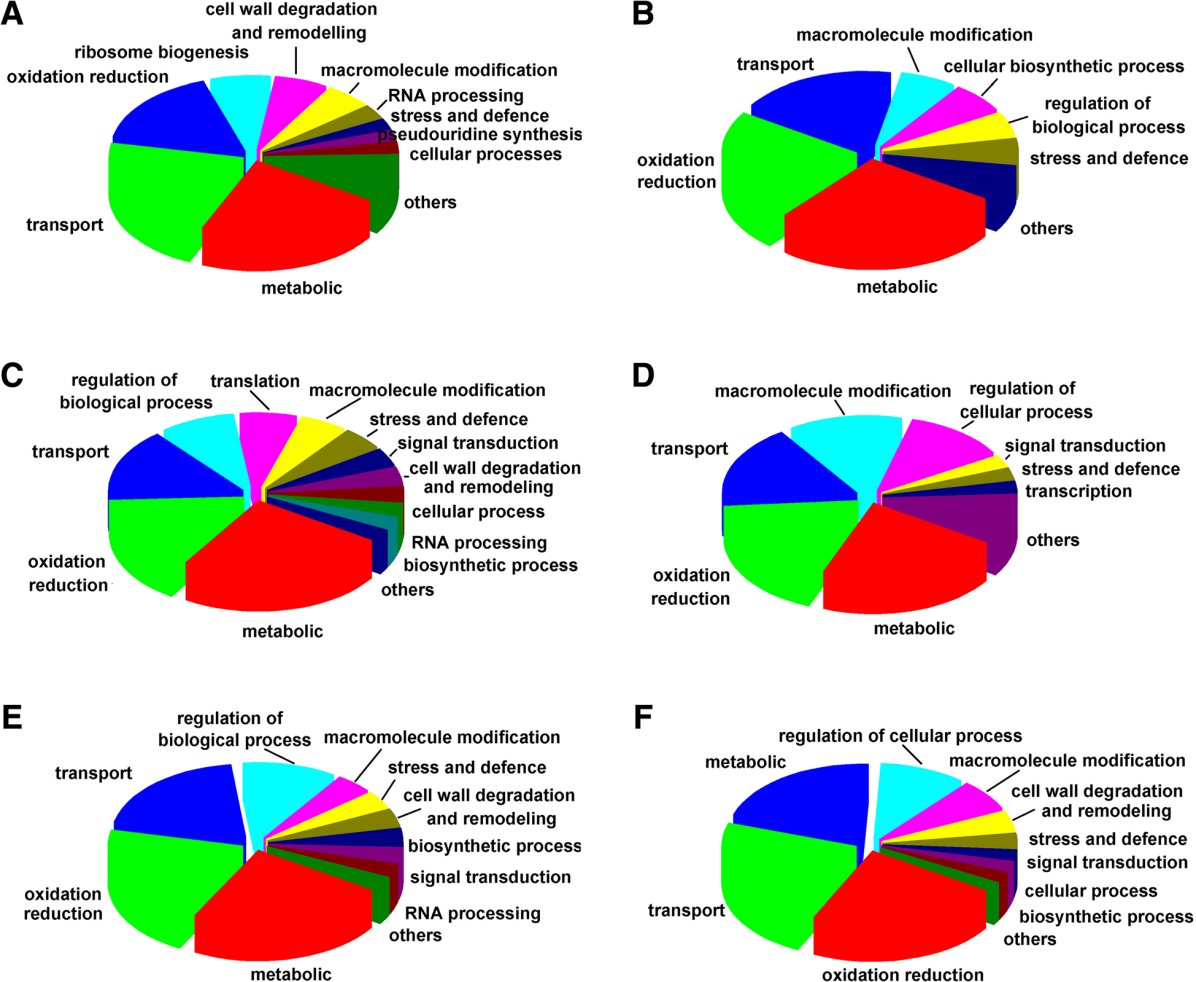
Categories of DEGs either unique to the high virulence strain or common to the both strans based on GO analyses for biological processes. **a** Up-regulated DEGs common to both strans. **b** Down-regulated DEGs common to both strains. **c** Uniquely up-regulated DEGs at 24 hai. **d** Uniquely down-regulated DEGs at 24 hai. **e** Uniquely up-regulated DEGs at 120 hai. **f** Uniquely down-regulated DEGs at 120 hai. DEG with proportions lower than 2% and DEGs without known biological process are not listed

Analysis of DEGs common to all four *in planta* datasets identified 190 significantly up-regulated genes and 360 down-regulated genes (Fig. 5), which indicates those genes may play key roles in pathogenicity. The common up-regulated genes were grouped into 18 Biological Process-GO terms. Among them, ‘metabolic processes’ were most abundant (21.9%), which mainly includes genes related to carbohydrate and amino acid metabolism, such as genes encoding Fructose-1,6-bisphosphatase, 2-methylcitrate synthase, glycerol kinase, glucosidase, and homogentisate 1,2-dioxygenase. Genes related to cellular transport were the second most abundant (21.1%). The identified putative transporter DEGs mainly belong to the major facilitator superfamily (MFS) transporters (CL2581.Contig1_All, CL2581.Contig2_All, Unigene6735_All), ATPases (CL502.Contig6_All, Unigene7523_All, Unigene5867_All, Unigene3458_All) and amino acid transmembrane transporters (Unigene2367_All, CL244.Contig3_All, Unigene10225_All, Unigene8441_All), which may play an important role in the secretion of endogenous fungal pathogenic factors during the infection. Transcripts encoding proteins with roles in ‘oxidation-reduction process’ (e.g., dioxygenases, oxidases, reductase and dehydrogenases), ‘cell wall degradation and remodeling’ (e.g., glucosidase, chitinase, pectate lyase and endoglucanase), ‘ribosome biogenesis’ (e.g., ribosome biogenesis protein, and ACA ribonucleoprotein complex subunit 2), ‘stress and defence’ (e.g., oxidative stress, detoxification and other stress-related genes), and ‘macromolecule modification’ (e.g., methylation and phosphorylation) were also highly up-regulated in the two strains (Fig. 6a; Additional file 6). Moreover, we found the gene encoding *Fo* Secreted In Xylem (SIX) protein SIX11 (Unigene11126_All) in the overlapping DEGs databases, the expression of which was highly induced at 24 and 120 hai in both strains (log_2_ Ratio 9.7~11.7). Finally, 88 DEGs featured unknown biological processes (Additional file 6).

For the overlapping down-regulated DEGs with known biological functions, genes related to ‘metabolic’ process were most abundant (27.0%), followed by genes involved in ‘oxidation-reduction process’ (23.0%), ‘transport’ (18.9%), ‘macromolecule modification’ (6.8%), ‘cellular biosynthetic processes’ (6.8%) and ‘stress and defence’ (5.4%) (Fig. 6b). The expression of genes related to MFS transporter, glucose transporter, alkanesulfonate monooxygenase, hexokinase, cytochrome P450 protein and dehydrogenase was highly variable (Additional file 6).

All the overlapping DEGs with highly differential expression (Fold change ≥64) were mapped on the reference genomes to determine its distribution on the genome. 24.3% of the overlapping up-regulated DEGs were located on the LS (lineage specific) chromosomes, including 4 genes related to oxidation-reduction process, 3 associated with transport, one related to cell wall degradation and remodelling and 10 genes with no annotation (Additional file 7). One hundred one overlapping down-regulated DEGs were found on the LS genomic regions, mainly including genes associated with metabolic, oxidation-reduction and stress and defence (Additional file 7).

### Unique DEGs of InVir in planta database links to pathogenicity differentiation

DEGs unique to the infection process of each strain were compared, and we most focused on the unique DEGs of InVir, indicating changes of *Foc* resulting in the increase of virulence. Relative to WT, 286 and 366 DEGs were uniquely significantly up-regulated in InVir at 24 and 120 hai, respectively (Fig. 5a). For the DEGs with known biological process, genes related to metabolic process were most numerous at both time points (such as succinate dehydrogenase, beta-glucosidase, homocitrate synthase, cytochrome b-c1 complex subunit 7, carboxypeptidase, alkaline proteinase, acyl-CoA oxidase, acyl-CoA thioester hydrolase, and ribose-phosphate pyrophosphokinase). However, several carbohydrate metabolism genes related to oxidative phosphorylation (Unigene646_All, Unigene10555_All and Unigene10554_All) were only found to be up-regulated at 24 h and may provide a good source of energy for the conidial germination and growth during early infection. A 15.8 and 22.2% of the uniquely up-regulated DEGs were linked to oxidation-reduction processes at the same time point, including NADH-ubiquinone oxidoreductase, 1,4-Benzoquinone reductase, peroxiredoxin, prephenate dehydrogenase, alcohol dehydrogenase, malate dehydrogenase, formate dehydrogenase, prostaglandin-endoperoxide synthase, tRNA-dihydrouridine synthase and cytochrome c549. A 15.2 and 18.0% of the up-regulated DEGs were involved in transport at 24 and 120 h, respectively, including ABC transporter, sodium efflux P-type ATPase, MFS transporter, sugar transporter, mitochondrial import inner membrane translocase, polyamine transporter and hypothetical protein classes. A 8.2 and 12.0% of the uniquely up-regulated DEGs were related to regulation of biological processes (e.g., translation initiation factor, 1,4-Benzoquinone reductase, Zinc finger protein, elongation factor 1-beta, SKN7 protein, programmed cell death protein, and transcription factor prr1) at the same time points. A 5.8 and 4.8% at 24 and 120 h, respectively, were involved in macromolecule modification (e.g., methyltransferase, octanoyltransferase, oligosaccharyl transferase, ubiquitin-conjugating enzyme, cell cycle control protein, dihydrouridine synthase, pseudouridylate synthase and serine/threonine-protein kinase psk1). Similarly, a 4.7 and 3.6% were related to stress and defence (e.g., formamidopyrimidine-DNA glycosylase, peroxiredoxin, catalase-peroxidase and prostaglandin-endoperoxide synthase) at 24 and 120 h, respectively. Some genes related to signal transduction, translation, and cell wall degradation and remodeling were also found at both time point, which included beta-glucosidase, mannosidase, exoglucanase, arabinosidase, xylanase, SKN7 protein and RNA polymerase. Moreover, 150 and 219 of the specifically upregulated DEGs featured unknown biological functions at 24 and 120 h, respectively (Fig. 6c, e; Additional file 8).

Meanwhile, 492 and 214 DEGs were uniquely down-regulated in InVir at 24 and 120 hai (Fig. 5b). A large fraction of these genes - 59.0 and 61.7% respectively- had no Biological Process annotation. Among the DEGs with known biological functions, transcripts related to metabolic process (22%) were dominant, followed by genes encoding oxidation-reduction process related-enzymes (17.9%), genes linked to transport (17.0%), genes involved in macromolecule modification (13.8%) and regulation of biology process (11.5%) at the early stages (Fig. 6d; Additional file 7). At 120 hai, transporters (23.0%) and oxidation-reduction processes (23.0%) represented the majority of classified proteins, followed by metabolic process (20.7%), regulation of biology process (10.3%), and macromolecule modification (6.9%) (Fig. 6f; Additional file 8).

From the the unique DEGs with over 64 change fold change compared to vegetative growth, 20 up-regulated genes in InVir at 24 hai were located on LS regions. These included 2 genes coding transposases (CL2135.Contig4_All and CL361.Contig32_All). At 120 hai expressed DEGs related to transport, regulation of biological process, and oxidation-reduction process also proved located on LS chromosomes (Additional file 9).

### Transposons may contribute to the adaption to hosts

During the expression studies *in planta*, 11 genes with annotation of transposition were differently expressed (Table 3). All 11 genes proved to be located on the lineage specific genome region, and most of them on the pathogenicity chromosome (Additional file 10). Four genes (CL1809.Contig3_All, CL119.Contig2_All, CL1911.Contig1_All and CL1911.Contig2_All) were up-regulated in both WT and InVir. Only two genes (CL126.Contig4_All and CL730.Contig6_All) with annotation of transposase were down-regulated in both strains. In addition, four genes (Unigene13826_All, CL361.Contig32_All, Unigene14252_All, CL2135.Contig4_All) were highly up-regulated in InVir during the whole infection period, however they were down-regulated in the weakly virulent strain WT at 24 hai. Except for Unigene13826_All, the expression levels of the other genes annotated with transposase function were all over 50 fold increased. In general, transposition genes in InVir were more active than in WT.

**Table 3:** DEGs annotated with function of transposition

GeneID	InVir 0 h vs 24 h			InVir 0 h vs 120 h			WT 0 h vs 24 h			WT 0 h vs 120 h			Blast nr	BP
	log_2_ Ratio	*R* ^a^	*P* ^b^	log_2_ Ratio	*R*	*P*	log_2_ Ratio	*R*	*P*	log_2_ Ratio	*R*	*P*		
CL1911. Contig2_All	3.54	Up	0.8	4.52	Up	0.8	2.57	Up	0.8	4.06	Up	0.8	transposase-like protein	–
CL1809. Contig3_All	8.35	Up	0.7	9.24	Up	0.8	10.12	Up	0.9	8.8	Up	0.8	transposase	DNA integration
CL119. Contig2_All	4.1	Up	0.8	3.96	Up	0.8	6.07	Up	0.8	6.12	Up	0.8	restless-like transposase	–
CL1911. Contig1_All	3.73	Up	0.8	3.69	Up	0.8	1.34	Up	0.7	2.63	Up	0.8	transposase-like	–
CL126. Contig4_All	−1.65	Down	0.6	−1.9	Down	0.6	−9.11	Down	0.8	−3.47	Down	0.7	putative transposase	–
CL730. Contig6_All	−0.83	Down	0.5	−2.47	Down	0.7	−0.7	Down	0.5	−9.65	Down	0.9	transposase	–
Unigene 13826_All	2.07	Up	0.6	7	Up	0.9	−3.42	Down	0.3	7.51	Up	0.9	retrotransposase	DNA integration
CL361. Contig32_All	9.88	Up	0.9	9.38	Up	0.9	−6.01	Down	0.5	2.42	Up	0.6	transposase	–
Unigene 14252_All	10.02	Up	0.9	7.31	Up	0.6	−5.4	Down	0.4	−5.37	Down	0.4	transposase	–
CL2135. Contig4_All	10.3	Up	0.9	6.83	Up	0.6	−6.77	Down	0.6	2.93	Up	0.7	restless-like transposase	–
Unigene 1211_All	−4.12	Down	0.3	5.76	Up	0.9	−5.06	Down	0.4	5.66	Up	0.8	retrotransposase	DNA integration

^a^Up-Down-Regulation

^b^Probability

### Weighted gene co-expression network analysis

**Fig. 7:**
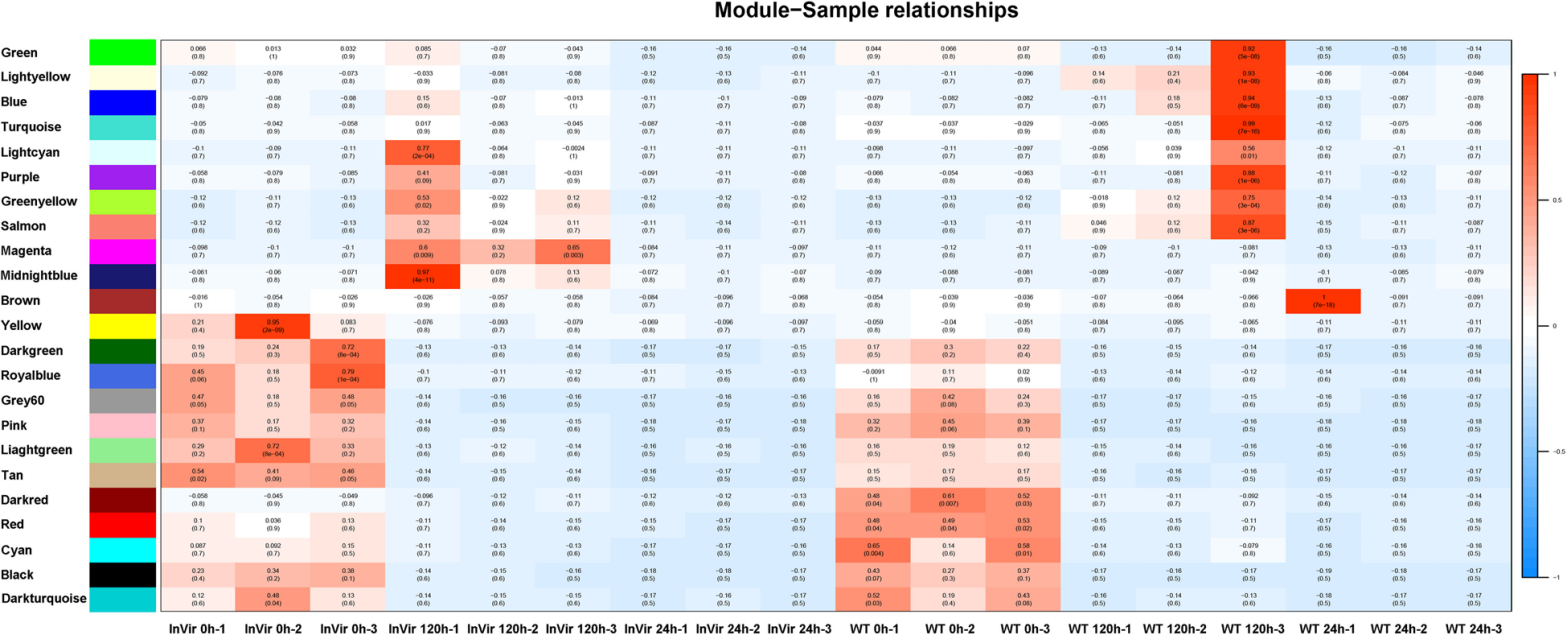
Weighted Gene Co-expression Network Analysis (WGCNA) of the transcripts changes in the highly (InVir) and weakly (WT) virulent strains. Module trait correlation analysis showed that six modules were correlated with the high virulence of *Foc* after infection

To screen for specific DEGs related to virulence, the data of the two strains during infection were subjected to weighted gene co-expression network analyses (WGCNA). A total of 1,247 transcripts were assigned to 23 co-expression modules, named after randomly assigned colors (Fig. 7). Six of them were relevant to highly virulent strain InVir responses (Fig. 7) as their eigengene was associated with the development of disease.

Here, two modules (‘midnightblue’ and ‘magenta’) were identified for further analysis. GO enrichment analysis was applied to investigate the function of transcripts in these two modules. In module ‘midnightblue’, several biological processes related to *Foc* virulence were enriched, including ‘metabolic process’ and ‘oxidation-reduction process’, ‘cell wall degradation and remodelling’ and ‘stress and defence’ (Additional file 11). In total, 14 genes belonged to this module, including exoglucanase (eg. CL2393.Contig1_All and CL2393.Contig2_All), prostaglandin-endoperoxide synthase 1 (eg. Unigene686_All and Unigene7471_All) and some hypothetical proteins were uniquely up-regulated in InVir (Additional file 11). In the ‘magenta’ module, both ‘metabolic process’ and ‘oxidation-reduction process’ GO terms were also overrepresented (Additional file 11). There were 22 genes were uniquely up-regulated in highly virulent strain InVir strain. However, most of the uniquely up-regulated genes in this module were hypothetical proteins (Additional file 11).

### Quantitative reverse transcription PCR validation

**Fig. 8:**
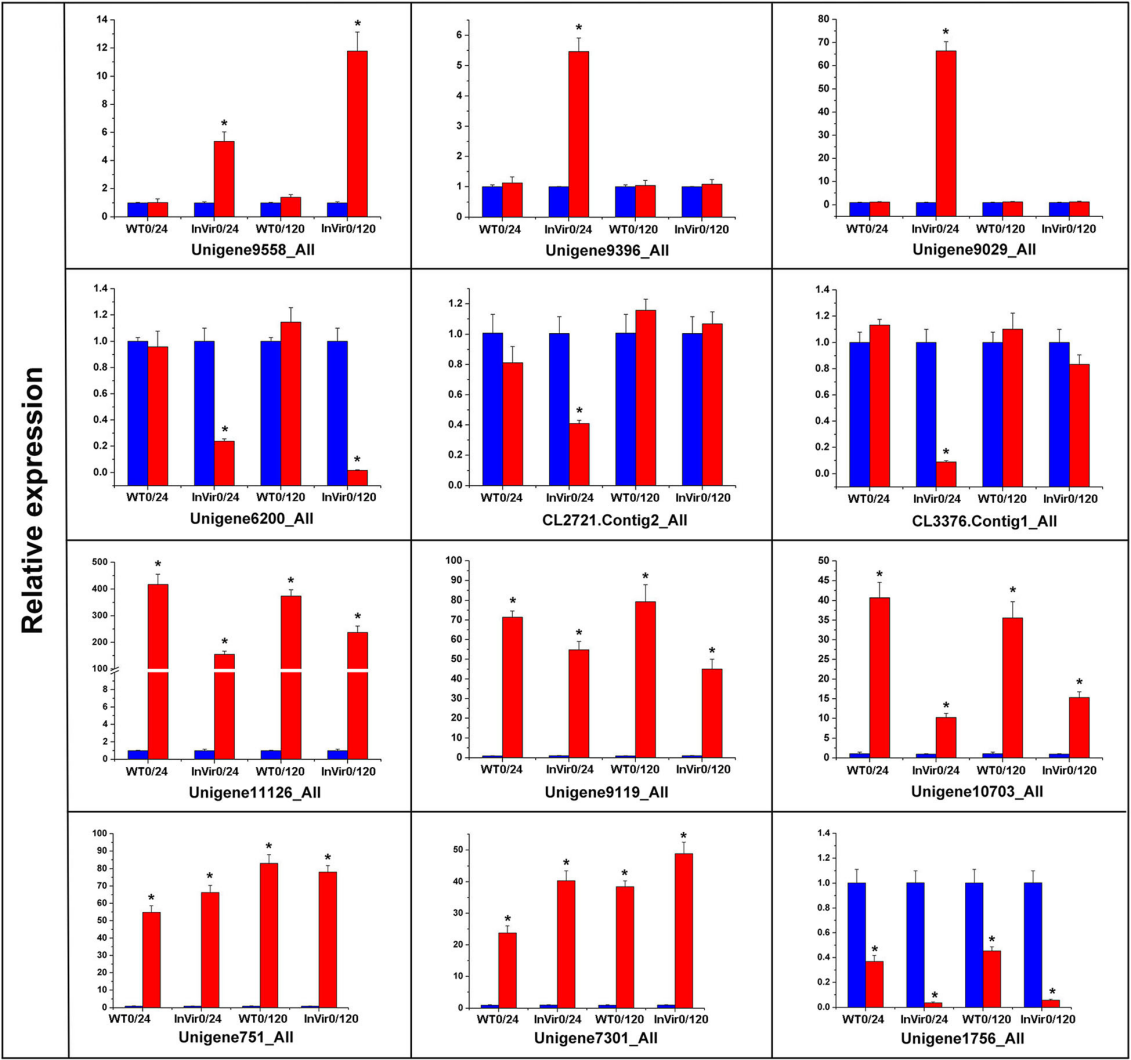
Verification of observed expression levels of differentially expressed genes of *Foc* under induction of cucumber in the transcriptome analysis, using quantitative reverse transcription PCR. The relative expression levels of 12 unigenes (Unigene 9558_All, Unigene9396_All, Unigene9029_All, Unigene 6200_All, Unigene9119_All, Unigene11126_All, Unigene10703_All, Unigene751_All, Unigene7301_All, Unigene1756_All, CL2721. Contig2_All, CL3376. Contig1_All) were determined in vitro and *in planta*. The bars in blue represent vegetative growth in vitro, and red represent infection of cucumber; W and I represent the weakly (WT) and highly (InVir) virulent strains, respectively; Numbers after the letters represent the sample time points (hours). 0 h represents the strains grown on media. Error bars indicate the standard deviation of three replicates

To confirm the reliability of the transcriptome analyses, the expression levels of a set of genes were validated by quantitative Reverse Transcription PCR (qTR-PCR). Twelve DEGs were selected that based on their expression levels could be divided into four groups (Fig. 8). Group 1 included DEGs upregulated during the infection of cucumber in the highly virulent strain InVir, which encoded a putative fumarate reductase, NADH-ubiquinone oxidoreductase and ATP-binding cassette. In group 2, DEGs were specifically down-regulated in InVir and the tested genes encoded a Vitamin H transporter, zinc finger protein and protein transport protein sec-13. DEGs encoding *six11*, aldose 1-epimerase, aldehyde dehydrogenase, murein transglycosylase, and a peroxisomal were placed in the third group, and were commonly upregulated during the whole infection by both strains. In group 4, an acyl-CoA dehydrogenase-encoding gene was downregulated in both the strains. The expression levels of all tested genes detected by qRT-PCR analyses were consistent with the results of transcriptome sequencing, indicating that the DEGs derived from the transcriptome during colonisation of cucumber were accurate and reliable, and hence suitable for further investigation of the genes associated with pathogenicity in *Foc*.

## Discussion

To better understand the mechanisms of pathogenicity and virulence variation of *F. oxysporum* f. sp. *cucumerinum* strains, a comparative transcriptomic analysis of a high and mild virulence strain was conducted during their infection of cucumber roots. Genes potentially related to pathogenicity and virulence differentiation were subsequently identified.

The host-pathogen interaction during several complex and crucial processes is important for the establishment of infection, especially during the initial infection stage [24]. A weakly virulent strain of *F. oxysporum* f. sp. *phaseoli* had a slower colonization and infection rate than the high virulent strain [10]. Hence, to determine the early stage of invasion, a molecular marker *GFP* was inserted into the weakly virulent strain WT to observe the infection process on cucumber roots. Based on the observation, samples from 24 and 120 hai were selected to represent the whole infection process at an early stage and at a later stage to construct the transcriptome of *Foc* colonization on the cucumber roots. To overcome the constraint of low *Fo* RNA abundance in cucumber root tissues early in infection, high coverage *in planta* RNA-Seq was conducted, and coupled with the pathogen’s vegetative (in vitro: 0 h) transcriptome to identify genes differentially expressed upon host infection. Finally, the mapping rates of *Foc* RNAs ranged from 1.12 to 5.92%. The mapping rates of the mild virulence isolate WT were significantly lower than the high virulence isolate InVir, especially at 120 hai, suggesting the colonization of the InVir was higher, and it was consistent with the virulence of the two isolates [8].

From the transcriptome of *Foc*, large numbers of DEGs between vegetative and *in planta* growth conditions were identified in both strains, especially at the early stage of infection. We deduced that upon the induction by the host plant, the *Foc* isolates change their life style from saprophytic to parasitic: numerous genes in the fungus related to infection are induced, while many genes involved in vegetative growth on Armstrong medium are repressed.

For the DEGs up-regulated in the both strains, the largest groups of expressed genes were significantly enriched for metabolic, transport and oxidative processes, which are similar to the results for the transcriptomes analysis of *F. oxysporum* f. sp. *cubense* and *F. oxysporum* f. sp. *medicaginis* during colonization of their respective hosts [9, 22]. However, the next abundant group was different from the *F. oxysporum* f. sp. *cubense* datasets that was enriched for primary/cellular/nitrogen compound metabolic and biosynthetic processes. In our study and the *F. oxysporum* f. sp. *medicaginis* datasets, they were followed by host cell wall and membrane degradation, supporting the speculation that these differences may potentially be due to differences in root tissue or cell structure between a dicot and monocot host [22].

In both mild and high virulence strains, genes encoding cell wall degrading enzymes (CWDEs), such as beta-glucosidase, feruloyl esterase, chitinase and pectate lyases were significantly up-regulated at the two time points and in accordance with other *Fo* studies [25–28]. This expression pattern might be related to hyphal growth behavior: at the early stage of infection, hyphae digest cell walls to penetrate and facilitate intercellular elongation, and at a later stage extend along plant cortex and epidermis (internaly and externally), and finally form expanding networks at 120 hai.

Screening for unique DEGs in virulence-enhanced variant InVir is significantly revealing that the molecular regulation mechanism for virulence differentiation of *Foc* under selective pressure by the host. Compared to the weakly virulent strain, genes involved in metabolism, including carbohydrate, lipid, and amino acid metabolism, were specifically upregulated in the high virulence strain, where they may provide the energy for the fungal growth, development and other process. In particular, several carbohydrate metabolic genes related to oxidative phosphorylation, were exclusively detected in the 24 hai DEGs dataset. When carbohydrate in the media becomes limiting, *Saccharomyces cerevisiae* can switch from glycolytic to oxidative phosphorylation metabolism [29]. We deduced that during early plant infection the energy metabolic of InVir had a similar adjustment to get more energy, and thus had a more rapid growth and stronger pathogenicity.

Some DEGs related to transport were also uniquely expressed in InVir, especially ATPase, inorganic ion and amino acid transport. Similar results were found in high virulent strains of *Curvularia lunata* and *F. oxysporum* f. sp. *conglutinans* [9, 30]. Transporters in plant pathogenic fungi play an important role in secretion of endogenous fungal pathogenic factors such as toxins, and in protection against exogenous plant defense compounds such as phytoalexins [31, 32]. Specific expression of these genes may aid InVir in its stronger virulence.

Some genes linked to post-translational modification (PTM) including protein phosphorylation, glycosylation and methylation were also enriched in the uniquely DEGs database, (eg. serine/threonine-protein kinase psk1, oligosaccharyl transferase and methyltransferase). PTM plays a significant role in a wide range of cellular processes, including cell cycle, growth, and signal transduction pathways [33]. For example, deletion of the N-glycosylation enzyme α-1,6-mannosyltransferase FoOCH1 from *F. oxysporum* f. sp. *cubense* reduced cell wall integrity and affected virulence on banana plants [34]. Deletion of the gene *bik3* encoding O-methyltransferases resulted in total loss of bikaverin synthesis in *F. fujikuroi* [35]. In addition, a number of genes without known function were significantly differentially expressed as well, implying that they might be linked with interesting functions during infection or virulence variation. However, the specific functions of the genes need further study.

The 11 genes linked with a transposition function were very active during infection of the host. All of these DEGs were located on the accessory genome, and most of them on the pathogenicity chromosome. It has been found that transposons are more active in lineage specific regions than core regions in *F. oxysporum* f. sp. *cepae* and *Verticillium dahlia* [20, 36, 37]. Among these DEGs with transposition function, beside 6 DEGs up- and down-regulated in both the mild as well as high virulence strain, there were 4 DEGs highly up-regulated only in virulence-increased isolate InVir at 24 hai, and there were no DEGs only up-regulated in WT. Generally, up-regulation happened more in InVir than WT, suggesting that the transposable elements are more active in the isolate with higher virulence. This suggests transposons contribute to the adaption to the host pressure. Effector genes tend to be distributed in regions enriched in transposable elements that may result in increased mutagenesis of effectors [38]. Very few effectors were actually identified in the transcriptome, which may be due to the high variability of effectors. In addition, using a de novo assembly, and also using transcripts as the reference gene sets may resulted in a reduction in the amount of identified effectors. Probably, novel effectors could be identified from the numerous hypothetical proteins. Further research on how transposons adjust the functional genes involved in infection, especially effectors associated with virulence, would help us to understand the fast adaptive evolution of this pathogen.

WGCNA was used to analyze the virulence-related genes during the highly and mildly virulent strains’ infection. Two expression modules were significantly associated with the high virulence, which included some uniquely DEGs in InVir, eg, genes related to metabolic, oxidation-reduction, cell wall degradation and remodelling and stress and defence. However, most of the DEGs in these two modules were hypothetical proteins.

## Conclusion

By a comparative transcriptome analysis of the mild and high virulence *Foc* strains during their infection of cucumber host, a series of DEGs that might be associated with virulence were identified. This study provides a first insight into the mechanisms underlying virulence differentiation of *Foc* and greatly improves our current understanding of Fusarium wilt pathogen molecular responses during infection of cucumber.

## Methods

### Isolates

The mild virulence strain foc-3b (WT, strain number: ACCC39326) and a virulence-enhanced variant Ra-4 (InVir) obtained by four serial passages of WT through a resistant cucumber cultivar were used in this study [8]. Both strains are maintained at − 80°C in 30% glycerol in the Biocontrol of Soilborne Diseases Laboratory of the Institute of Plant Protection, Chinese Academy of Agricultural Sciences (CAAS).

### Plant cultivars

The susceptible cucumber cultivar *Cucumis sativus* L. cv. Zhongnong No. 6 (ZN6) was provided by the Institute of Vegetables and Flowers, CAAS.

### Construction of GFP-marker strain of *Foc*

In order to visualize colonization and infection process in vivo, the strain WT was marked with GFP. The plasmid pSC003 carrying GFP and antibiotic resistance gene *G418* was transformed into WT strain using PEG-CaCl_2_ mediated transformation as described previously [39]. Transformants emerging on PDA plates with 200 mg/ml G418 were picked up, and the morphology and GFP expression of the transformants were examined. After inoculation on PDA for five generations, putative transformants were selected by fluorescence microscopy (BX61, Olympus, Tokyo, Japan).

The virulence of mutants with stable fluorescence was compared to wild type strain WT according to Huang et al. [8]. A mutant gWT with stable fluorescence and similar virulence with wild type properties was selected and used to inoculate cucumber plants to determine suitable time points for transcriptome profiling.

### Colonization of cucumber roots by *Foc*

Cucumber seeds were disinfected in an oven at 68 °C for 3 h, and then placed on Murashige and Skoog (MS) medium [40] in glass jars (dia. 9 cm) and incubated in a growth chamber at 28 °C with a photoperiod of 16 h light/8 h dark for 2 weeks.

The gWT was inoculated into Armstrong liquid medium [41] and incubated at 28 °C on a shaking table at a speed of 180 r/min. After 3 days, the liquid culture was passed through a 30 μm sterile mesh to remove hyphae. The filtrate was centrifuged at 6000×*g* for 10 min and washed with sterile distilled water for three times to remove residual medium. The conidia were resuspended in sterile distilled water and used for inoculation of the cucumber seedlings.

A soil mixture consisting of vermiculite, peat, pearlite and sand (1:1:1:3, v/v/v/v) was autoclaved at 121 °C for 1 h and mixed together with gWT conidia suspension with a final inoculum density of 10^5^ spores/g soil. The inoculated soil was put in plastic pots (dia. 6 cm). Two-week-old cucumber seedlings growing on the MS medium were transplanted into the pots, one seedling per pot, and 10 plants per replicate. A soil mixture inoculated with sterile distilled water was taken as the control. The pots were placed randomly in a growth chamber with a constant temperature of 26 °C and a photoperiod of 16 h light/8 h dark.

Observations of the cucumber roots were made at 24, 36, 48, 72, 96 and 120 h after transplanting. Three to five plants were carefully taken out of the pots and the roots were gently rinsed with tap water. Then the whole roots were mounted in drops of water on glass slides, and the fluorescence was detected by a Confocal Laser Scanning Microscope (LSM 880, Zeiss, Jena, German) using an excitation wavelength of 522 nm.

### Inoculation and sampling

The inocula of the highly virulent and mild virulence strains were prepared and inoculated using the same method as described above. After inoculation, the cucumber seedlings were maintained in a growth chamber with a photoperiod of 16 h light/8 h dark at 26 °C. For each strain, root tissue was collected from 5 plants and pooled per replicate at 24 and 120 hai, respectively. For *Foc* in vitro samples, mycelia were grown in Armstrong liquid medium on a shaking table at a speed of 180 r/min for 3 days at 26 °C. After centrifugation, mycelia and spores were harvested and supernatant discarded. The six samples were frozen immediately in liquid nitrogen and kept at − 80 °C. Three replicates were used per treatment.

### RNA extraction, cDNA libraries construction and Illumina sequencing

Total RNAs from the mycelia and infected root tissues were extracted using Trizol reagent (Invitrogen, Carlsbad, USA) according to the manufacturer’s instruction, and residual genomic DNA was removed by using DNase I (TaKaRa, Dalian, China). The concentration and quality of the total RNA were validated using a micro-spectrophotometer (SimpLiNano, Cambridge, UK) and an Agilent 2100 Bioanalyzer (Agilent Technologies, Santa Clara, USA). PolyA mRNA was isolated from the total RNA using magnetic beads harbouring oligo (dT) and broken into short fragments using fragmentation buffer in a Thermomixer (Eppendorf, Hamburg, Germany). Reverse transcription was conducted and cDNA fragments were synthesized after purification, end reparation, addition of adenine to the 3′ end, and adapter connection. Fragments were amplified to construct cDNA libraries. After quantity and quality monitoring, 18 cDNA libraries were sequenced using the Illumina HiSeq 4000 platform (Illumina Inc., CA, USA) at the Beijing Genome Institute.

### Transcriptome assembly and analysis

Clean reads were obtained for the 18 libraries by removing disqualified reads containing adapters, poly-Ns, and low-quality bases from the raw reads with Trimmomatic [42]. Clean reads were submitted to the NCBI sequence read archive, Accession Numbers: SRR6793782–6793799. De novo transcriptome assembly of the six mycelium samples was carried out using the short reads assembly program Trinity [43], which served as a reference gene set of *Foc*. The high quality reads were assembled into contigs, and then integrated to obtain unigenes. The quality of the assemblies was evaluated by some criteria including total number of the contigs produced, mean contig length and N50. BUSCO (v3.0.2), a pipeline used to accurately annotate core genes in eukaryotic genomes, was used to determine the completeness of the assemblies [44]. BLASTX alignment (E-value < 10^− 5^) was conducted to determine the sequence direction and to predict the protein coding regions. The CDSs were extracted from the unigenes, and those sequences that did not match any BLAST results were predicted using the ESTScan program [45]. Annotation of the unigenes was performed using NR, Swiss-Prot, GO (Gene ontology), KEGG (Kyoto encyclopedia of genes and genomes) and COG (Clusters of orthologous groups) databases. The program Blast2GO was used to obtain GO annotations regarding Cellular Component, Biological Process and Molecular Function of the unigenes [46], and the Blastall software was used to predict and classify the COG and KEGG pathway-associated unigenes [47, 48].

### Identification of DEGs and bioinformatics analysis

The fungal transcripts were filtered by mapping all the clean reads to the reference assembled transcriptome of *Foc* using HISAT and Bowtie2 software [49, 50]. The quality of the alignment was calculated at the Q20, Q30 and unique gene mapping ratio. The genes were further analyzed for changes in expression levels.

Transcript levels of the two *Foc* strains during infection of cucumber at two different time points were quantified using RNASeq by Expectation Maximization (RSEM) and calculated using the fragments per kb per million fragments (FPKM) method [51]. The NOISeq software package was used to screen DEGs. DEGs were identified using expression varied more than 4-fold and the probability divergence was higher than 0.8 as the threshold [52]. The functions of identified DEGs were then investigated by GO and KEGG pathway enrichment analysis. The number of DEGs of the GO and KEGG terms of the two isolates at 24 and 120 hai were compared by chi-square test using SAS 9.1.3 (SAS Institute Inc., Cary, NC, USA). Identification of unique or overlapping genes within the DEG datasets and the generation of Venn diagrams were determined using Draw Venn Diagram http://bioinformatics.psb.ugent.be/webtools/Venn/ (accessed 12–01-16). The genomes of *F. oxysporum* f. sp. *radicis-cucumerinum* and *F. oxysporum* f. sp. *lycopersici* have been separated in accessory genome and core genome and the two genomes were used as reference genome [18, 53]. Whether the DEGs are located on accessory or core genome was analyzed by mapping them on the reference genomes by using the method previously reported by Armitage and coworkers [36].

### Weighted gene co-expression network analysis

Weighted Gene Co-expression Network Analyses (WGCNA) were used to analyze the global coexpression network of the weakly and highly virulent strains during infection [54]. First, all the normalized values for each transcript from each time point in InVir and WT samples were collected to identify modules that had different expression patterns. Then, the networks were created using the method described by Langfelder and Horvath [54], and the resulting modules were merged based on the correlations of module eigengenes.

### Reverse transcription quantitative PCR verification

To confirm the reliability of the transcriptome data, a total of 12 genes that are similarly up- or down-regulated in both strains or uniquely up- or down-regulated in the high virulent strain were selected from the transcriptome of *Foc* for quantification in the strains at different times post inoculation using quantitative reverse transcription PCR. Primers of those genes were designed using the software Primer Premier 5.0 (Table 4), and their specificity was determined using PCR with the following program: 94 °C for 3 min; 30 cycles of 94 °C for 1 min, 60 °C for 30 s and 72 °C for 30 s; followed by 72 °C for 10 min. Total RNAs extracted from thallus and root tissue samples were reverse transcribed into cDNA using the cDNA FastQuant RT Kit (Tiangen, Beijing, China). The expression levels of the 12 DEGs were assayed using SYBR Premix Ex Taq II (TransGen, Beijing, China) with *EF1α* serving as internal reference gene [10] in an IQ 5 multicolour real-time PCR detection system (Bio-Rad, CA, USA). The reaction was performed in a 25 μl system containing 2 μl of 4-fold diluted cDNA, 1 μl of 10 μM forward and reverse primer, 12.5 μl of SYBR Premix, and 8.5 μl of RNase-free water. The qRT-PCR conditions were as follows: initial denaturation at 95 °C for 2 min, followed by 40 cycles of 95 °C for 10 s and 60 °C for 30 s. Fluorescence values were collected every 0.5 °C from 60 °C to 85 °C for 81 cycles to check non-specific amplification. Relative expression levels were calculated using the 2^–ΔΔCt^ method [55], where ΔΔCt = (Ct target - Ct *EF1α*) _infected sample_ - (Ct target - Ct *EF1α*) _hyphae sample_. Three replicates were conducted for each sample.

**Table 4:** Primers of *Foc* used for quantitative reverse transcription PCR

Gene	Predicted function	Primer (5′-3′)	Product length (bp)
Unigene9558_All	Putative fumarate reductase	F:CGAGCTCCTTACCGGTCATC	108
		R:TCCCCATTCGCCTTGTTCTC	
Unigene9396_All	NADH-ubiquinone oxidoreductase	F:AGGAACACTCGCATTACCCG	108
		R:AAAGTACTGCCTCGACGCAA	
Unigene9029_All	ATP-binding cassette	F:TGCGGATTTTGTGGTGCTTG	137
		R:AGGAGCAAGCTGCCATTGAT	
Unigene6200_All	Transporter	F:TAGAGGGTCTGGACTTGCGA	150
		R:GCGTCGCCACATCTTCAATC	
CL2721.Contig2_All	Zinc finger protein	F:GATCCTGCAACGTCGCAATC	129
		R:TCGCCATGTCGGATAAGCTC	
Unigene11126_All	Six11	F:GGCTTCGGGTCTCGTTTACA	199
		R:TCGTACGCAATTCATCCCGT	
Unigene9119_All	Aldose 1-epimerase	F:CTGATCGACGACCAGTACGG	104
		R:GGAACACCTCCAAGATGGGG	
Unigene10703_All	Aldehyde dehydrogenase	F:AGGGCAGAGAGAGGAGTCTG	151
		R:ACTACACCCGATCTGAGCCT	
Unigene751_All	Transglycosylase	F:CTTAACCATCTCGGCGTCGA	104
		R:GTATCCACCGATCCCACGTC	
Unigene7301_All	Peroxisomal	F:ACCAGCGAGAATGTCAGCAA	118
		R:TCTCATCGGCGAACAAACCA	
CL3376.Contig1_All	Transport protein	F:CCCAAGTACGGCAACATCCT	129
		R:GTTGACCGAGGCCTTGTGTA	
Unigene1756_All	Acyl-CoA dehydrogenase	F:GAGTGTTGGTTCCAGAGCGA	196
		GAGATCGTTGTCGCGAGGAT	
*EF1α*	–	F:CATCGGCCACGTCGACTCT	144
		R:AGAACCCAGGCGTACTTGAA	

### Statistical analysis

The expression levels of DEGs were analyzed using SAS 9.1.3 statistical software (SAS Institute Inc., Cary, NC, USA). For comparisons of the means of each treatment, *t* tests were used and *P*-values < 0.05 were regarded as significant.

## Additional files

Additional file 1: Statistics of the assembly quality of the transcriptome of *Foc* in vitro samples. (XLSX 13 kb)

Additional file 2: Transcriptome completeness of transcripts quantified through 290 universal single-copy orthologs using BUSCO. (EMF 79049 kb)

Additional file 3: The GO annotation of the unigenes of *Foc* in vitro samples. (EMF 2373 kb)

Additional file 4: KEGG analysis of the transcriptome of *Foc* in vitro samples. (XLSX 16 kb)

Additional file 5: All DEGs of the two strains *in Planta* compared to vegetative growth. (XLSX 944 kb)

Additional file 6: Highly overlapping up- and down-regulated DEGs in both strains. (XLS 306 kb)

Additional file 7: The genomic distribution of the overlapping DEGs with over 64 fold change in the two strains. (XLS 205 kb)

Additional file 8: Uniquely up- and down-regulated DEGs in high virulent strain InVir at 24 and 120 hai, respectively. (XLS 352 kb)

Additional file 9: The genomic distribution of the uniquely DEGs with over 64 fold change in InVir . (XLS 322 kb)

Additional file 10: DEGs annotated with transposition function. (XLS 38 kb)

Additional file 11: Genes of two co-expression modules in InVir. (XLSX 40 kb)

## Data Availability

The RNA-Seq data 18 samples of two *Foc* isolates are available in the Sequence Read Archive (SRA) repository of NCBI with the accession numbers SRR6793782–6793799.

## Abbreviations

COG: Clusters of orthologous groups
CWDE: Cell wall degradation enzyme
DEG: Differentially expressed gene
*Foc*: *Fusarium oxysporum* f. sp. *cucumerinum*
GFP: Green fluorescent protein
GO: Gene ontology
hai: h after inoculation
KEGG: Kyoto encyclopedia of genes and genomes
MS: Murashige and Skoog
NR: Non-redundant Protein Sequence Database
NT: Nucleotide Sequence Database
PTM: Post-translational modification
